# Volumetric changes of porcine collagen matrix and free gingival grafts for soft-tissue grafting to increase the width of keratinized tissue around dental implants: a retrospective clinical study

**DOI:** 10.1186/s40729-024-00575-6

**Published:** 2024-11-12

**Authors:** Ausra Ramanauskaite, Katharina Melissa Müller, Carla Schliephake, Karina Obreja, Amira Begic, Iulia Dahmer, Puria Parvini, Frank Schwarz

**Affiliations:** 1 Department of Oral Surgery and Implantology, Goethe-University Frankfurt, Carolinum, Frankfurt am Main, Germany; 2 Faculty of Medicine, Institute of Biostatistics and Mathematical Modelling, Goethe-University Frankfurt, Frankfurt am Main, Germany

**Keywords:** Dental implants, Free gingival graft, Keratinized mucosa, Soft tissue grafting, Three-dimensional analysis

## Abstract

**Aim:**

To compare three-dimensional changes of aporcine derived collagen matrix (CM) and free gingival grafts (FGG) for increasing keratinized tissue (KT) at dental implants over a 24-month follow-up period.

**Materials and methods:**

This retrospective study enrolled 25 patients exhibiting 41 implants with deficient KT width (i.e., < 2 mm) who underwent soft tissue augmentation using either CM (11 patients/15 implants) or FGG (14 patients/26 implants). The primary outcome was tissue thickness change (mm) at treated implant sites between 1- (S0), 12- (S1), and 24-months (S2). Secondary outcome was the changes of KT width over a 24-month follow-up period.

**Results:**

Dimensional analyses from S0 to S1 and from S0 to S2 revealed a mean decrease in tissue thickness of -0.05 ± 0.35 mm and − 0.31 ± 0.41 mm in the CM group, and − 0.23 ± 0.38 mm and − 0.22 ± 0.81 mm in the FGG group, with no significant differences found between the groups (S0-S1: *p* = 0.14, S0-S2: *p* = 0.58). Within S1 and S2, the CM and FGG groups displayed comparable tissue thickness reduction (CM: -0.32 ± 0.53 mm, FGG: -0.02 ± 0.21 mm; *p* = 0.07). The FGG group exhibited a significantly greater KT gain 24-months compared to the CM group (CM: 1.50 ± 1.14 mm, FGG: 4.04 ± 1.65 mm; *p* < 0.001).

**Conclusions:**

CM and FGG were associated with comparable three-dimensional thickness changes over a period of 24 months. A significantly wider KT band could be established in the FGG group.

**Graphical Abstract:**

**Figure:**
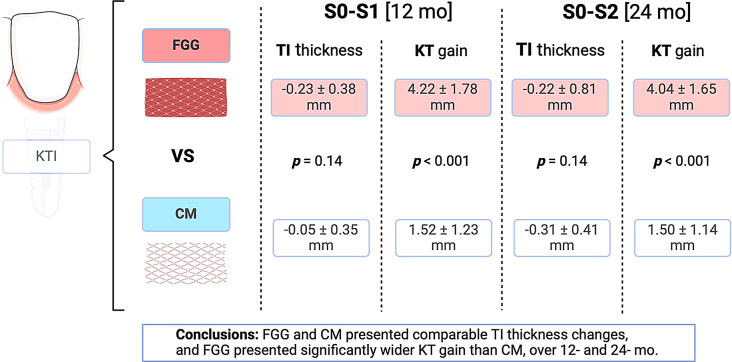
Graphical abstract of the study. KTI: keratinized tissue increase; FGG: free gingival graft; CM: collagen matrix; TI: tissue

**Supplementary Information:**

The online version contains supplementary material available at 10.1186/s40729-024-00575-6.

## Introduction

Extensive clinical data underlined the importance of peri-implant soft-tissue characteristics for the maintenance of peri-implant tissue health and stability [1–3]. In particular, a reduced amount of keratinized mucosa (i.e., KT < 2 mm) was found to be related with increased prevalence of peri-implantitis, higher plaque accumulation, soft-tissue inflammation, mucosal recession, marginal bone loss, and greater patient discomfort [3, 4]. Consequently, soft-tissue grafting aimed at establishing a band of KT is recommended at implant sites where insufficient amount of KT associates with recurrent inflammation of peri-implant mucosa, discomfort upon brushing, increased soft-tissue recession, lack of attached mucosa, or a shallow vestibular depth [3, 4]. In fact, surgical establishment of KT resulted in considerably improved peri-implant tissue conditions, defined by lower plaque indices, probing depth (PD) values, and marginal bone stability compared to nonaugmented implant sites [5].

An apically positioned flap in combination with the free gingival autogenous graft (FGG) is currently considered a standard of care intervention to establish KT at dental implants [3, 6]. Though the procedure leads to highly predictable outcomes, it is also associated with numerous disadvantages, such as postoperative patients’ morbidity, surgical complications related to the donor site, and high shrinkage rates of the graft [3, 7, 8]. Thus, substitute materials of xenogenic origin constitute an alternative to autogenous tissues and were shown to lead to highly promising clinical results when compared to autogenous grafts [9]. As such, according to the current clinical recommendation, xenogenic soft-tissue substitutes may be preferred over autogenous grafts in patients with limitations in the donor area or when a limited amount of KT is required [3].

The thickness of peri-implant mucosa is another soft-tissue feature essential for the stability of peri-implant tissues [6, 10, 11]. More specifically, implant sites featuring thin soft-tissues (i.e., < 2 mm) were found to undergo a higher initial bone remodeling, show inferior esthetic outcomes, and possess a higher risk for peri-implant diseases [5, 10, 12]. One recent randomized clinical study (RCT) reported on comparable volumetric soft-tissue thickness changes at implant sites treated with the FGG and porcine collagen matrix [13]. The findings were, however, limited to a 6-month observation period, thus indicating the need for medium- to long-term follow-up data [13]. Therefore, the aim of the present retrospective study was to assess and compare three-dimensional (3D) changes occurring at dental implant sites treated with the FGG or collagen matrix over a follow-up period of 24 months.

## Materials and methods

This retrospective analysis included the data of 25 patients that initially participated in a two-arm RCT aimed at assessing volumetric changes at implants sites treated with a porcine collagen matrix (CM) or FGG for establishing KT at dental implants [13]. The initial study population consisted of 34 patients (17 in the CM group and 17 in the FGG group). Of them, eight (5 in the CM group and 3 in the FGG group) did not attend the follow-up appointments, and one patient in the CM group moved to another country; thus, 25 patients (11 in the CM group and 14 in the FGG group) were feasible for inclusion.

All patients were fully or partially edentulous and exhibited at least one dental implant with a deficient KT width (i.e., < 2 mm) at the vestibular aspect. The patients were treated between December 2020 and February 2022 at the Department of Oral Surgery and Implantology, Goethe University, Frankfurt, Germany, and were enrolled in a regular yearly maintenance program. The study was in accordance with the Helsinki Declaration of 1975 (revised in August 2018) and approved by the Ethics Committee of the Goethe University (No. 20–541). The study was retrospectively registered in the German Clinical Trials Register (DRKS00025237).

### Inclusion criteria

For patient selection, the following inclusion criteria were defined:

1. minimum age of 18 years,
2. edentulous or partially edentulous patients who had undergone dental implant surgery at grafted and/or non-grafted (i.e., pristine) sites,
3. patients treated with FGG or CM at dental implants featuring KT width of < 2 mm at the vestibular aspect either at the time of second-stage surgery (i.e., 3 to 5 months following submerged healing) or implants in function,
4. presence of peri-implant tissue health,
5. adequate oral hygiene as evidenced by plaque index (PI) < 1,
6. patients who attended 12- and 24-months’ follow-up appointment.

## Exclusion criteria

The exclusion criteria considered patients who presented with:

1. uncontrolled diabetes (HbA1c > 7),
2. autoimmune and/or inflammatory diseases of the oral cavity,
3. active periodontal disease,
4. pregnant or lactating women,
5. smokers (≥ 10 cigarettes per day), and
6. malpositioned implants,
7. patients who did not attend 12-months follow-up appointment.

## Treatment procedures

**Fig. 1:**
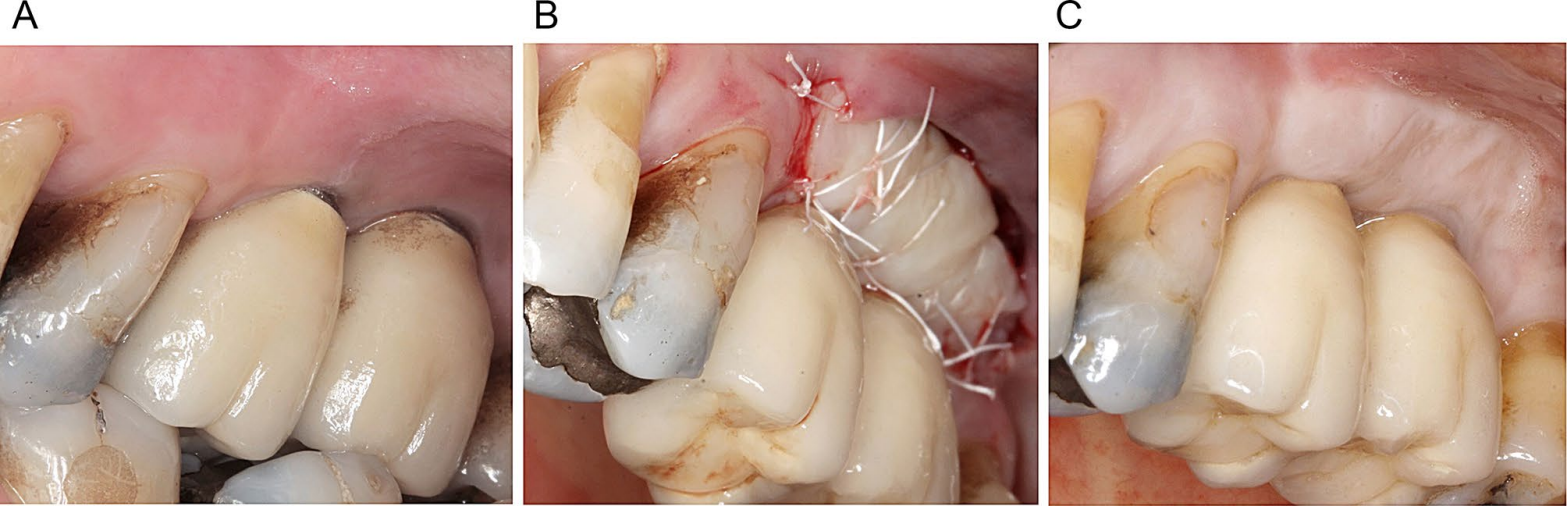
Surgical procedure in the free gingival graft (FGG) group: **A** preoperative intraoral view showing a lack of keratinized tissue at implants 16 and 17. **B** postoperative view depicting the fixation of FGG at the recipient site. **C** intraoral view of the surgical site after 12 months

**Fig. 2:**
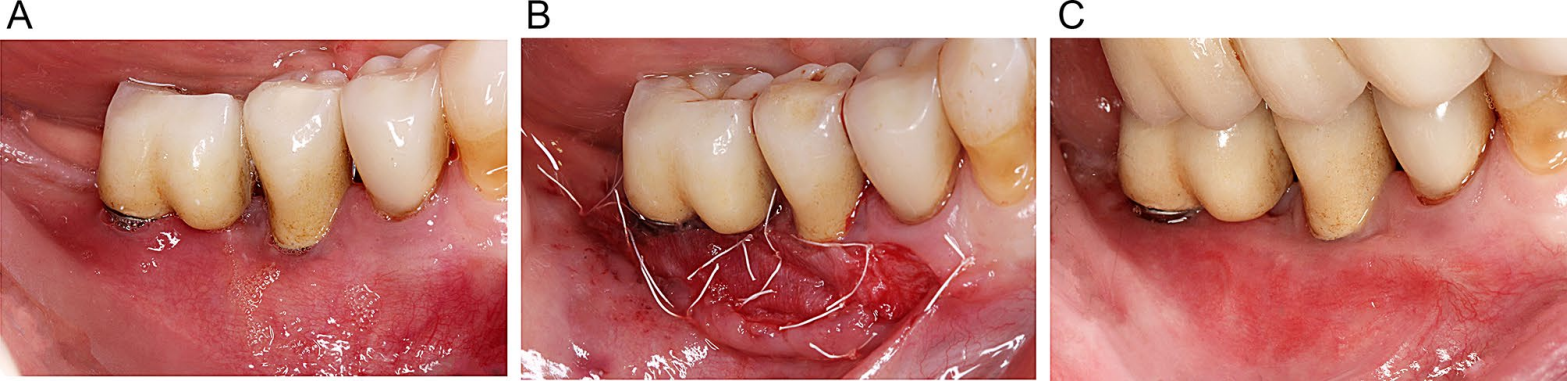
Surgical procedure in the collagen matrix (CM) group: **A** preoperative intraoral view indicating a lack of keratinized tissue at implants 35 and 36. **B** Postoperative view showing the fixation of CM at the recipient site. **C** Intraoral view of the surgical site after 12 months

All surgical procedures were performed by four calibrated experienced clinicians (AR, KO, AB, PP). Following the administration of local anesthesia (2% articaine, 1:100.000 epinephrine), healing abutments of appropriate dimensions were inserted at the implant sites undergoing a simultaneous second-stage surgery. Subsequently, the recipient bed was prepared using a 15-stainless steel blade by performing a horizontal split-thickness incision at the mucogingival junction (MGJ) on the buccal aspect of the implants. In the absence of KT at the recipient area, the entire mucosa at the implant’s buccal aspect was raised. The mucosa was apically positioned and fixed to the periosteum with 4/0 non-resorbable PTFE monofilament sutures (Cytoplast PTFE, Osteogenics Biomedical, Lubbock, USA). In the FGG group, a 1.0- to 1.5-mm-thickness FGG was harvested from the hard palate between the first premolar and the first molar and 2 mm from the gingival margin of the adjacent teeth. To retain the clot, the donor site was sutured with a 4/0 non-resorbable PTFE monofilament. The FGG was positioned and fixed to the periosteum at the recipient bed with interrupted and mattress sutures with 4/0 non-resorbable PTFE material (Fig. 1). In the collagen matrix group (CM group), a porcine collagen matrix (Geistlich Mucograft, Geistlich Biomaterials, Wolhusen, Switzerland; CM) was used. The matrix was trimmed to the required dimensions and fixed to the periosteum with interrupted and mattress sutures with 4/0 non-resorbable PTFE monofilament (Fig. 2). Ten days after surgery, the sutures were removed and the sites were rinsed with 0.12% chlorhexidine digluconate. At implant sites where surgeries were performed simultaneous to implant uncovering, the prosthetic treatment was performed 3 months after surgery.

## Primary outcome – volumetric tissue thickness changes

The primary outcome was defined as 3D tissue thickness changes (mm) at the vestibular aspect of treated implant sites after a 24-month follow-up period (“Soft- tissue augmentation core outcome set and measurements (STA-COSM) core outcome areas and domains for soft-tissue augmentation) [14]. To assess this outcome, intraoral digital scans of the treated area were acquired using an intraoral scanner (3 Shape Trios move, Germany GmbH) after a healing period of 1 month (S0), 12 months (S1) and 24 months (S2). To enable accurate superimposition of the scans taken at multiple points, caution was taken to capture reproducible fixed reference points (i.e., adjacent teeth/implants, anatomical structures).

**Fig. 3:**
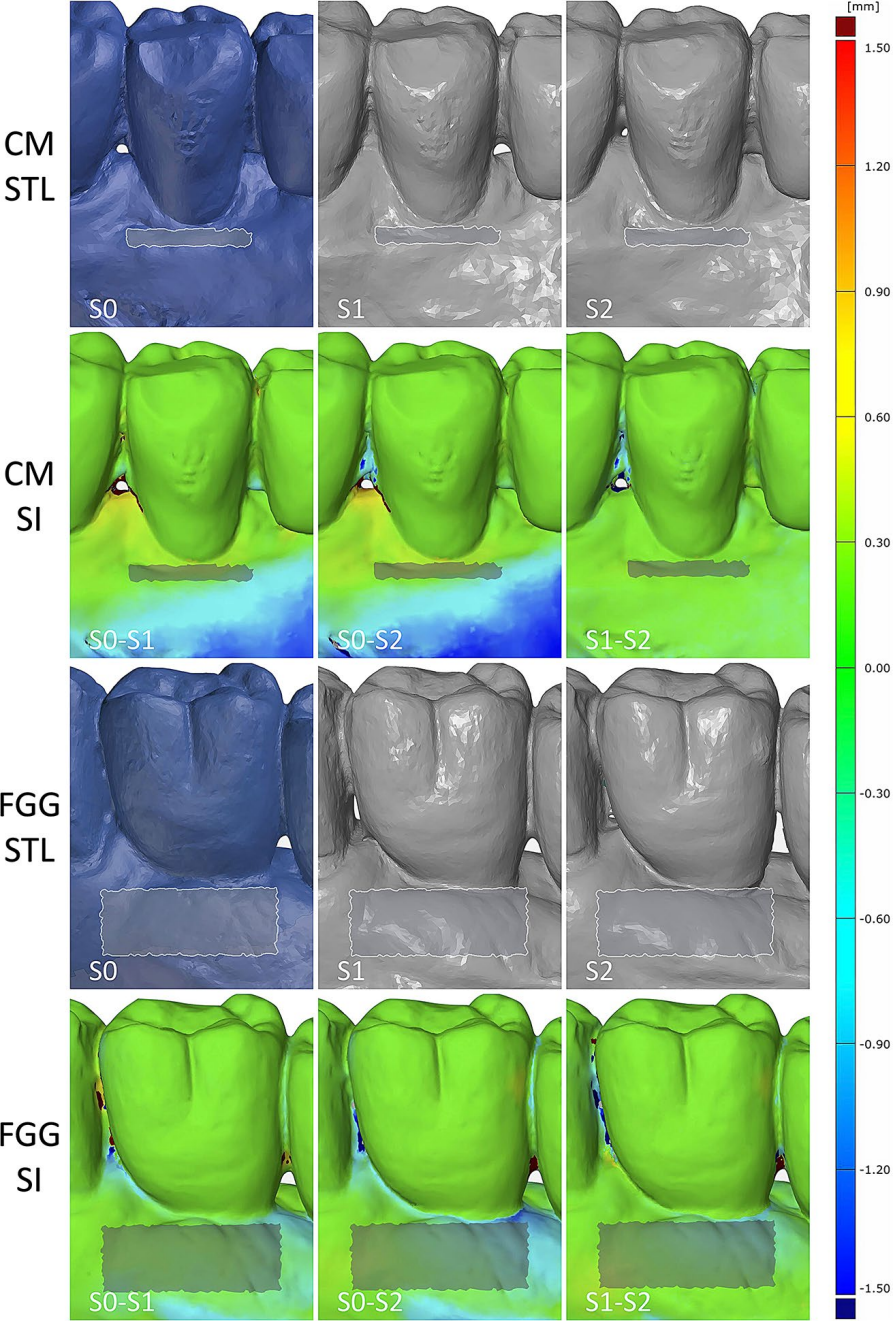
The illustration of two cases displaying the assessed volumetric measurements at the region of interest (ROI) in the CM and FGG groups at different follow-up periods. SI- superimposition. CM – collagen matrix group, FGG – free gingival graft group

The scanned files were saved as the Standard Tessellation Language (STL) files and exported into Zeiss Inspect (Optical 3D, 2023, Zeiss Company, Braunschweig) software. The S0, S1 and S2 digital models were simultaneously aligned by manually by selecting at least eight reproducible points (i.e., neighboring teeth, rugae palatinae). To account for the grafts’ surface shrinkage, the vertical extension of the standardized region of interest (ROI) was estimated by subtracting the KT values measured preoperatively from the KT measurements assessed at S2. The horizontal extension of ROI corresponded to the highest mesial-distal width of the prosthetic restoration (i.e., equator area) (Fig. 3).

The thickness changes occurring in the standardized ROIs were recorded from S0 to S1, from S0 to S2, and from S1 to S2. For each superimposed digital model, the minimum and maximum deviation and the arithmetic mean with its standard deviation were recorded. Two experienced and calibrated examiners (KM and CS) performed all measurements. Each analysis was performed in duplicate. Prior to the start of the analysis, an inter-examiner calibration was performed to determine the reproducibility of the measurements. The calibration when repeated measurements of 5 scans presented an intraclass correlation coefficient between 0.86 and 1.

## Secondary outcome – changes in KT width

Secondary outcome was the changes in KT width over 24-months. To assess this outcome, the KT width was measured with a periodontal probe prior to the surgery from the mucosal margin to the MGJ at the mid-vestibular aspect of the implant. The MGJ was determined by the color contrast between the KT and the alveolar mucosa. Two calibrated investigators (KM and CS) assessed the KT measurements at 12- and 24-months.

### Power calculation

With the included sample size (11 and 14 patients in the CM and FGG groups, respectively), an effect size of d = 1.4 can be recognized by a t-test with a power of 80% at a significance level of alpha 1.67% (obtained through Bonferroni correction due to three t-tests being performed, one for each time point) (BiAS for Windows). Assuming a standard deviation of 0.3 (Ramanauskaite, Obreja, Müller, Schliephake, Wieland, Begic et al., 2023) for the tissue thickness change allows to recognize minimal mean difference of 0.42 between the groups.

### Statistical analysis

The statistical analysis was performed using R-Studio and R softwares (packages “epiDisplay”, “nlme”). Mean values, standard deviations, medians, minimums/maximums, and 95% confidence intervals (95% CI) for primary and secondary outcomes are reported. The analysis was carried out at implant- and patient-levels. For the patient-level analysis, in patients with more than one implant, the mean values of the multiple implants were used in the analyses. Data was checked for normality using the Shapiro-Wilk-test.

For the patient-level analysis, Wilcoxon-Mann-Whitney-U-tests were used to compare the primary and secondary outcomes between the groups at the three time points. To account for multiple testing, a Bonferroni correction was considered leading to a significance level of 1.67% for this analysis.

At the implant-level, multiple linear regressions with mixed effects were used to assess the differences between the groups for the surface thickness change over time, KT width values at baseline, 12 and 24 months, and changes in KT width at the different time points. Nested random effects (the patient and the implant) were employed considering the treatment group (CM and FGG) and the time points as fixed effects. In order to adjust for baseline KT values as a confounder, this variable was also considered as a fixed effect in the regression.

A multiple linear regression analysis with mixed effects was conducted to evaluate the influence of the treatment approach (i.e., CM or FGG) and the change in KT width from S0 to S2, S1 to S2, and S0 to S2 on volumetric change at the treated implant sites.

## Results

In total, 25 patients with 41 implants were enrolled in this retrospective analysis. Eleven patients (9 female and 2 male; mean age65.76 ± 12.36 years) with 15 implants were assigned to the CM group. The remaining 14 patients (5 female and 9 male; mean age: 65.22 ± 8.41 years) with 26 implants were assigned to the FGG groups, of which all attended the 12- and 24-motnhs follow-up visits.

Table 1 represents patients´ demographic data and implant site characteristics. Five patients/6 implants in the CM group and 9 patients/15 implants in the FGG group were subjected to soft-tissue grafting at second-stage surgery, and the remaining 6 patients/9 implants in the CM group and 5 patients/9 implants in the FGG group were treated after functional implant loading. Except for two implant sites (1 in the FGG group and 1 in the CM group), all surgeries were performed in posterior areas.

**Table 1:** The demographic data and implant site characteristics

	CM	FGG
Patient number (n)	11	14
Female/ male (n)	9/2	5/9
Age (mean ± SD/ median) (years)	65.76 ± 12.36/65.28	65.22 ± 8.41/65.39
Implants	15	26
Region		
Upper jaw anterior anterior^*^/posterior	0/4	1/7
Lower jaw anterior/posterior	1/10	0/18
Second stage surgery Implants in function	5 patients/6 implants 6 patients/9 implants	9 patients/17 implants 5 patients/9 implants

Anterior* - from canine to canine, CM – collagen matrix group, FGG – free gingival graft group, SD – standard deviation

### Dimensional assessments

**Fig. 4:**
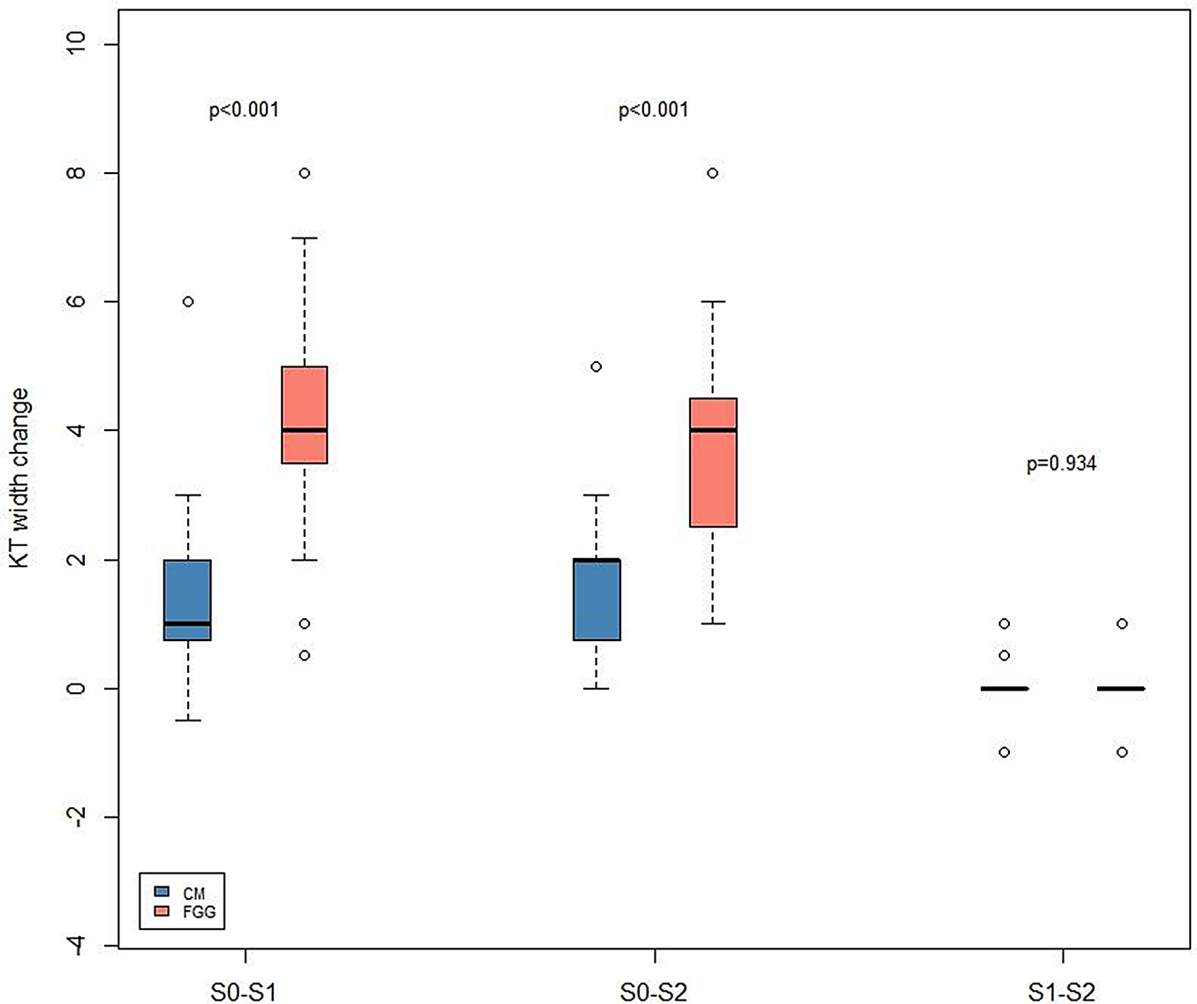
Box-plot depicting the assessed volumetric changes in the FGG and CM groups. Red bars – FGG group; blue-bars – CM group. CM – collagen matrix group, FGG – free gingival graft group. S0 – intraoral scan taken at after 1 month, S1 - intraoral scan taken at after 12 months, S2 - intraoral scan taken at after 24 months

Table 2 and Fig. 4 represent the dimensional changes assessed in both groups over a 24-month follow-up period.

**Table 2:** Thickness changes between analyzed time intervals over a 24-month follow-up period

	CM			FGG			
**Patient-level**	S0-S1* (11 patients)	S0-S2** (11 patients)	S1-S2*** (10 patients)	S0-S1* (11 patients)	S0-S2** (13 patients)	S1-S2*** (12 patients)	
Mean ± SD 95%-CI	0.05 ± 0.35 (-0.20; 0.29)	-0.31 ± 0.41 (-0.59; 0.04)	-0.32 ± 0.53 (-0.69; 0.06)	-0.23 ± 0.38 (-0.48; 0.03)	-0.22 ± 0.81 (-0.71; 0.27)	-0.02 ± 0.21 (-0.12; 0.15)	
**Implant-level**	**S0-S1** ^**§**^ (14 implants)	**S0-S2** ^**§§**^ (15 implants)	**S1-S2** ^**§§§**^ (14 implants)	**S0-S1** ^**§**^ (19 implants)	**S0-S2** ^**§§**^ (23 implants)	**S1-S2** ^**§§§**^ (22 implants)	
Mean ± SD 95%-CI	-0.05 ± 0.33 (-0.14; 0.24)	-0.36 ± 0.47 (-0.62; -0.10)	-0.37 ± 0.57 (-0.70; -0.05)	-0.18 ± 0.36 (-0.36; -0.02)	-0.09 ± 0.24 (-0.10; 0.11)	0.008 ± 0.24 (-0.10;0.0.11)	

CM – collagen matrix group, FGG – free gingival graft group, SD – standard deviation. S0 – intraoral scan taken at after 1 month, S1 - intraoral scan taken at after 12 months, S2 - intraoral scan taken at after 24 months

Between group comparisons – patient level: Wilcoxo-Mann-Whitney-U-Test: * *p* = 0.14** *p* = 0.58; *** *p* = 0.07

implant level: multiple linear regression with mixed effects: ^**§**^*p* = 0.169; ^§§^*p* = 0.725; ^§§§^: *p* = 0.285

Overall, within 1 and 24 months (S0–S2), implant sites in both the CM and FGG groups showed decreases in tissue thickness. In particular, S0 to S2 estimations revealed a tissue thickness reduction of − 0.31 ± 0.41 mm (patient-level analysis) and − 0.36 ± 0.47 mm (implant-level analysis) in the CM group, as well as − 0.22 ± 0.81 mm (patient-level analysis) and − 0.09 ± 0.24 mm (implant-level analysis) in the FGG group. Based on the patient- and implant-level analyses, differences between the groups were not statistically significant (patient-level: *p* = 0.58, Wilcoxon–Mann–Whitney U test; implant level: *p* = 0.689, multiple linear regression with mixed models).

Within S0 and S1, a tissue thickness decrease of − 0.05 ± 0.35 mm (patient-level) and − 0.05 ± 0.33 mm (implant-level) was measured in the CM group. The corresponding measurements in the FGG group amounted to − 0.23 ± 0.38 mm (patient-level) and − 0.09 ± 0.24 mm (implant-level). The differences between the groups were not statistically significant (patient-level: *p* = 0.14, Wilcoxon–Mann–Whitney U test; implant-level: *p* = 0.169, multiple linear regression with mixed models).

Based on the patient-level analysis, between S1 and S2, tissue thickness reduction of -0.32 ± 0.53 mm and − 0.02 ± 0.21 was measured in the CM and FGG groups respectively, with no significant difference found between the groups (*p* = 0.07; Wilcoxon–Mann–Whitney U test). The corresponding estimations at the implant level were − 0.37 ± 0.57 mm (CM group) and 0.008 ± 0.24 mm, with no significant difference found between the groups (*p* = 0.265; multiple linear regression with mixed models).

### Clinical assessments

**Fig. 5:**
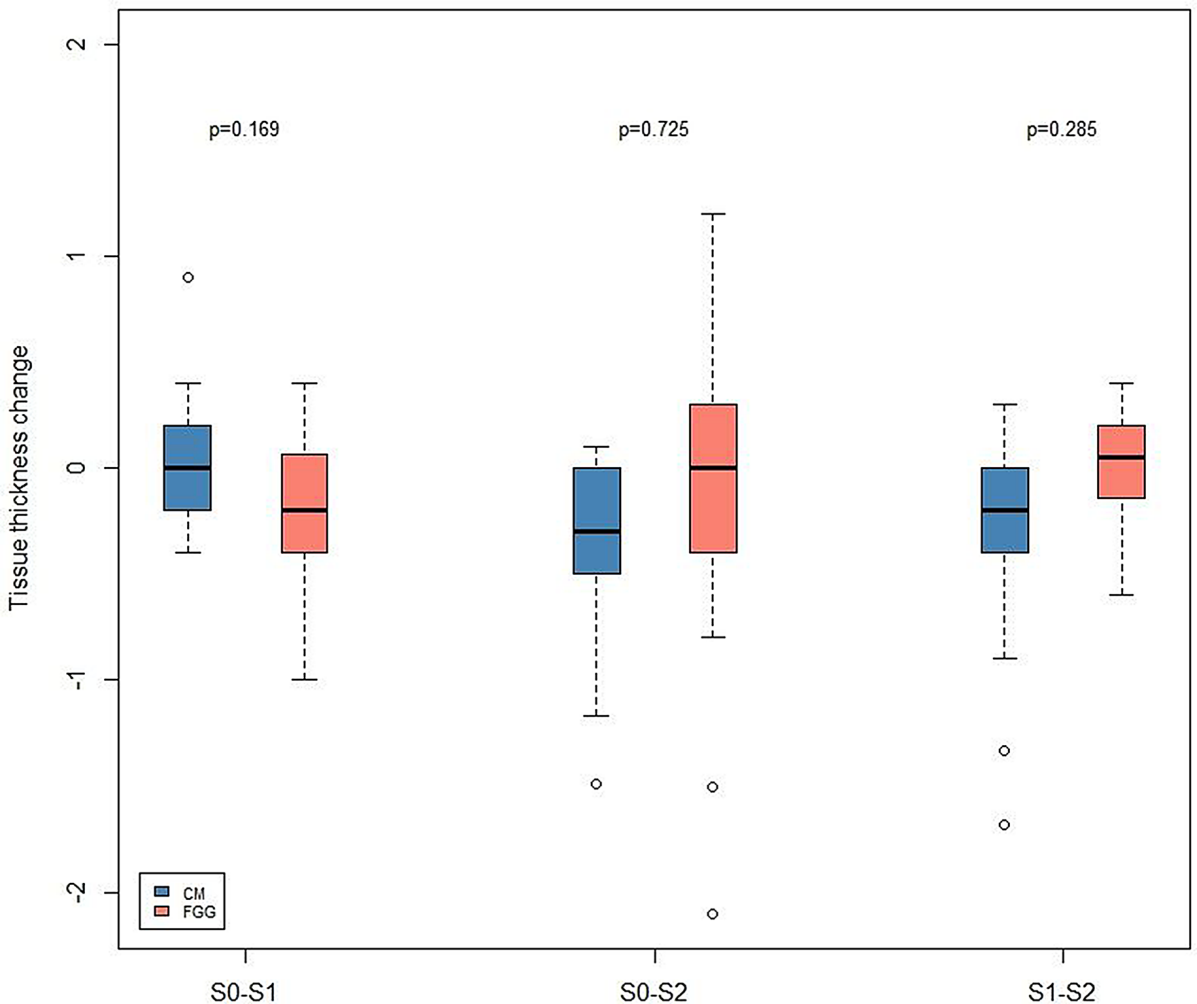
Box-plot illustrating the keratinized mucosa (KT) width changes over the investigation period in the FGG and CM groups. Red bars – FGG group; blue-bars – CM group. CM – collagen matrix group, FGG – free gingival graft group. S0 – intraoral scan taken at after 1 month, S1 - intraoral scan taken at after 12 months, S2 - intraoral scan taken at after 24 months

The KT measurements at different time points and the changes in KT width over the investigation period are presented in Tables 3 and 4, as well as in Fig. 5.

**Table 3:** The width of KT measured over a 24-month follow-up period

Patient-level	Baseline	12 months	24 months
**CM** mean ± SD 95%-CI	(11 patients) 0.95 ± 1.06* (0.24;1.06)	(11 patients) 2.45 ± 0.91** (1.85; 3.06)	(11 patients) 2.45 ± 0.91*** (1.85; 3.06)
**FGG** mean ± SD 95%-CI	(14 patients) 0.34 ± 0.68* (-0.05;0.73)	(14 patients) 4.56 ± 1.62** (3.63; 5.49)	(14 patients) 4.38 ± 1.48*** (3.52; 5.24)
**Implant-level**			
**CM** mean ± SD 95%-CI	(15 implants) 0.83 ± 1.03^§^ (0.26; 1.40)	(15 implants) 2.37 ± 1.42^§§^ (1.58; 3.15)	(15 implants) 2.40 ± 1.06^§§§^ (1.82; 2.98)
**FGG** mean ± SD 95%-CI	(26 implants) 0.37 ± 0.69^§^ (0.09; 0.64)	(26 implants) 4.15 ± 1.72^§§^ (3.65; 5.04)	(26 implants) 4.15 ± 1.49^§§§^ (3.55; 4.76)

CM – collagen matrix group, FGG – free gingival graft group, SD – standard deviation

Between group comparisons: patient-level: Wilcoxon-Mann-Whitney-U test: * *p* = 0.09; ** p0.001; *** *p* < 0.001

implant level: multiple linear regression with mixed effects: ^§^*p* = 0.69; ^§§^*p* < 0.001; ^§§§^: *p* < 0.001

**Table 4:** Changes of KT width over a 24-month follow-up period

Patient-level	Change Baseline − 12 months	Change Baseline − 24 months	Change 12-months – 24 months
**CM** mean ± SD 95%-CI	(11 patients) 1.52 ± 1.23* (0.70; 2.25)	(11 patients) 1.50 ± 1.14** (0.73; 2.67)	(11 patients) -0.02 ± 0.48*** (-0.35; 0.30)
**FGG** mean ± SD 95%-CI	(14 patients) 4.22 ± 1.78* (3.19; 5.25)	(14 patients) 4.04 ± 1.65** (3.09; 4.99)	(14 patients) -1.18 ± 0.32*** (-0.36; 0.004)
**Implant-level**			
**CM** mean ± SD 95%-CI	(17 implants) 1.53 ± 1.47^§^ (0.77; 2.29)	(17 implants) 1.21 ± 1.61^§§^ (0.38; 2.03)	(17 implants) -0.32 ± 1.13^$$^ (-0.90; 0.26)
**FGG** mean ± SD 95%-CI	(26 implants) 3.98 ± 1.84^§^ (3.24; 4.72)	(26 implants) 3.79 ± 1.61^§§^ (3.14; 4.44)	(26 implants) -0.19 ± 0.49^$$^ (-1.39; 0.006)

CM – collagen matrix group, FGG – free gingival graft group, SD – standard deviation. S0 – intraoral scan taken at after 1 month, S1 - intraoral scan taken at after 12 months, S2 - intraoral scan taken at after 24 months

Between group comparison patient-level: Wilcoxon-Mann-Whitney-U test: * *p* < 0.001; ** *p* < 0.001; *** *p* = 0.274

Based on the patient-level analysis, after 12 and 24 months, mean KT measurements in the CM amounted to 2.45 ± 0.91 mm and 2.45 ± 0.91 mm, respectively. The respective measurements in the FGG groups were 4.56 ± 1.62 mm (12 months) and 4.38 ± 1.48 mm (24 months). The differences between the groups were statistically significant, favoring the FGG group after 12 and 24 months (12 months: *p* = 0.001; 24 months: *p* < 0.001; Wilcoxon–Mann–Whitney U test). According to the implant-level measurements, after 12 and 24 months, the mean KT width in the CM group was 2.37 ± 1.42 mm and 2.40 ± 1.06 mm, as well as 4.15 ± 1.72 mm and 4.15 ± 1.49 mm in the FGG group, favoring the FGG group (12 months: *p* < 0.001, 24 months: *p* < 0.001, multiple linear regression with mixed models).

As for the KT width changes, after 12 and 24 months, the mean KT gain in the CM group was 1.52 ± 1.23 mm and 1.50 ± 1.14 mm at the patient-level, as well as 1.53 ± 1.47 mm and 1.21 ± 1.61 mm at the implant-level. In the FGG group, after 12 and 24 months, the mean KT gain amounted to 4.22 ± 1.78 mm and 4.04 ± 1.65 mm at the patient level, and 3.98 ± 1.84 mm and 3.79 ± 1.61 mm at the implant level. After 12 and 24 months, the KT gain significantly favored the FGG group (patient level: 12 months: *p* < 0.001; 24 months: *p* < 0.001, Wilcoxon–Mann–Whitney U test; implant level: 12 months: *p* < 0.001; 24 months: *p* < 0.001).

At the patient- level, within 12 and 24 months, the KT width reduced by − 0.02 ± 0.48 mm in the CM group and by − 1.18 ± 0.32 mm in the FGG group (*p* = 2.74; Wilcoxon–Mann–Whitney U test). At the implant-level, the KT reduction between 12 and 24 months in the CM group was − 0.32 ± 1.13 mm, and–0.19 ± 0.49 in the FGG group, with no significant differences found between the groups (*p* = 0.935; multiple linear regression with mixed models).

According to the multiple linear regression analysis with mixed effects, a significant influence of the change in KT width upon the volumetric tissue thickness changes was found in the CM group between S0 and S1, pointing to an estimated increase in the tissue volume of 0.15 for every 1-mm increase in the KT change (*p* = 0.036). Between S0 and S2, and S1 and S2, no significant association among the changes in KM width and volume tissue in the CM group could be detected (*p* = 0.425, *p* = 0.983). In the FGG group, the change in KT width did not have an influence on the tissue thickness changes at all of the investigated time points (S0-S2: *p* = 0.421, S0-S1: *p* = 0.953, S1-S2: *p* = 0.440).

## Discussion

This retrospective analysis aimed at assessing the 3D changes in tissue thickness at implant sites that underwent soft-tissue grafting procedures to increase KT width using an apically positioned flap with either CM or FGG. Volumetric tissue thickness changes occurring after 12 and 24 months were compared to the tissue thicknesses assessed 1 month after the healing.

Overall, the findings of the present analysis pointed toward a comparable reduction in tissue thickness within 1 and 24 months in both groups, which at the patient-level analysis in the CM group amounted to − 0.31 mm, and − 0.22 mm in the FGG group. Between 1 and 12 months, a decrease in tissue thickness tended to be higher in the FGG group (–0.23 mm versus − 0.05 mm; patient-level analysis), whereas between 12 and 24 months, a greater tissue thickness loss was measured in the CM group (–0.32 mm versus − 0.02 mm; patient-level analysis). In corroboration are the estimations of the implant-level analysis that, between 12- and 24 months, revealed a higher tissue thickness loss in the CM group compared to the FGG group (–0.09 mm versus − 0.36 mm). Between 1 and 12 months, a decrease in tissue thickness was higher in the FGG group (–0.18 mm versus − 0.04 mm), whereas within 12 and 24 months, a higher reduction was registered in the CM group (–0.37 mm versus 0.008 mm), without significant differences detected between the groups at any investigation time point. The initial analysis of the same patient sample reported the 3D tissue changes occurring over a 6-month period in the FGG and CM groups [13]. In particular, within 1 and 6 months, implant sites in both the FGG and CM groups showed a reduction in tissue thickness, with a slightly higher tissue thickness decrease between 1 and 3 months documented in the CM group [13]. Between 3 and 6 months, comparable tissue thickness shrinkage was registered in both groups [13]. To the author’s best knowledge, to date, this is the only clinical analysis assessing and comparing the 3D tissue thickness changes at implant sites treated with FGG or CM.

As advocated in one previous RCT, which assessed one-dimensional tissue thickness changes based on the measurements obtained in the CBCT scans, compared to the preoperative tissue thickness, after 6 months, implant sites treated with FGG showed an increase in tissue thickness of 0.11 ± 0.06 mm, whereas reduction in tissue thickness was documented in the CM group (–0.19 ± 0.12 mm; intra-group comparison: *p* = 0.002) [15]. On the other hand side, another RCT, which measured one-dimensional tissue thickness by using an endodontic file with a rubber stop, reported on the gain in tissue thickness at implants treated with CM and FGG, which after 2 and 6 months significantly favored implant sites in the FGG group compared to the baseline (i.e., preoperative tissue thickness; preoperative – 2 months: FGG: 1.0 ± 0.3 mm, CM: 0.1 ± 0.4 mm, *p* < 0.001; preoperative – 6 months: FGG: 0.9 ± 0.5 mm, CM: 0.1 ± 0.5 mm; *p* = 0.003) [16]. Between 2 and 6 months, a slight tissue thickness decrease of 0.1 mm occurred in the FGG group, whereas tissue thickness remained stable in the CM group [16]. Contrary to this are the results addressed by another RCT, which used a probe with a rubber stop to assess tissue thickness alterations at implant sites augmented either with CM or FGG [17]. In particular, within 1 and 6 months, the CM group displayed a significantly higher decrease in tissue thickness compared to the FGG group (− 0.53 mm versus − 0.36 mm, respectively; *p* < 0.001) [17]. Nonetheless, it must be highlighted that the aforementioned studies were limited to a 6-month follow-up period and assessed one-dimensional assessments, which subsequently prevent any direct comparison with the present findings.

Upon further analysis of the present data set, a significantly higher mean KT gain after 12 and 24 months was obtained in the FGG group. Specifically, after 12 and 24 months, the mean KT gain in the CM group amounted to 1.52 and 1.50 mm at the patient-level analysis, and to 1.53 mm 1.21 mm at the implant-level analysis. In the FGG groups, after 12 and 24 months the mean gain in KT was 4.22 and 4.04 mm at patient level, as well as 3.98 and 3.79 mm at the implant-level, respectively. Keeping in mind the recent recommendations that define the width of < 2 mm of KT as insufficient, it appears that the use of CM may not allow to obtain the desired endpoint of establishing at least 2 mm of KT [3]. Based on the results of the initial analysis of the same patient sample, after 6 months, the mean KT gain in the CM and FGG groups was 1.47 ± 1.25 mm and 3.94 ± 4.90 mm at implant-level analysis, as well as 1.45 ± 1.13 mm and 4.52 ± 1.46 mm at patient-level analysis, respectively, significantly favoring the FGG group [13]. The aforementioned outcomes basically suggest that the KT width established after 6 months could be maintained throughout the 24-month period. It must be acknowledged that, to date, a majority of the existing clinical studies comparing the FGG and CM groups for KT establishment at dental implant sites are limited to a 6-month follow-up period and reported upon the mean KT gain of 3.73 to 4.47 mm at the implant sites treated with FGG, as well as 2.51 to 3.23 mm at the implant sites treated with CM [17, 18]. In contrast to our findings, after 12 months, one retrospective analysis indicated comparable KT gain in the FGG and CM groups (4.10 ± 1.16 mm and 3.37 ± 0.97 mm, prospectively) [19]. Furthermore, within 6 to 12 months, a significant decrease in KT width was documented in the CM group, whereas insignificant changes were measured in the FGG groups [19]. In the present analysis, between 12 and 24 months, tissue thickness reduction was documented in the CM and FGG groups, without significant differences noted between the two groups. In line with this are the results of another retrospective analysis that revealed a constant decrease in KT width in both the FGG and CM groups throughout a mean follow-up period of 2.3 years (FGG group) to 2.6 years (CM group), with a greater graft contraction observed in the CM group [20].

According to the current findings, it is important to note that, after 24 months, three implants in two patients in the CM group displayed no gain in KT width compared to the preoperative KT measurements, whereas all implant sites possessed an increase in KT width in the FGG group. The latter finding underlines the importance of the case selection for the decision on the surgical approach, as autogenous grafts may be favored at sites with complete absence of KT, and soft-tissue substitutes could be considered when a limited amount of KT is needed [3].

When interpreting the present findings, it must be acknowledged that due to the fact that the software estimated the sutures present in the postoperative scan to the surface thickness calculation, an intraoral scan taken after 1 month was used as a baseline. The latter did not permit assessment of the overall tissue thickness changes, including those occurring within the first 4 weeks. In addition, for the volumetric and clinical measurements, loaded and unloaded implants have been pooled, which might have relevantly altered the accuracy of the ROI as well as the clinical measurements, particularly at implant sites treated during the second-stage surgery. Furthermore, implant sites with either a complete absence of KT and those with a reduced width (< 2 mm) were merged into the analysis, which might have considerably affected the dimensional alterations and changes in KT width at dental implant sites treated with FGG and CM. It is worth noticing that in the present analysis, we did not investigate the surface shrinkage of the grafted site during the investigation period due to the similarities of the grafted area in terms of tissue structure and color in the CM group compared to the surrounding tissues, which subsequently led to difficulties in demarcating the grafted site in the respective group. A relatively small sample size enrolled in the analysis might have further affected the obtained outcomes. Finally, it is important to underline that one of the major limitations of the present analysis is the lack of the assessment of the clinical parameters defining peri-implant tissue health status, such as plaque values, bleeding on probing, and probing pocket depth. The latter did not permit to relate the surgical procedure implemented for the KT establishment and the maintenance of peri-implant tissue health.

Within the limitations of the present study, it was concluded that CM and FGG were associated with comparable 3D thickness changes over a period of 24 months. A significantly wider KT band could be established in the FGG group.

## Electronic supplementary material

Below is the link to the electronic supplementary material.

Supplementary Material 1

## Data Availability

No datasets were generated or analysed during the current study.
